# Gianotti-Crosti syndrome: a case report of a teenager*

**DOI:** 10.1590/abd1806-4841.20164410

**Published:** 2016

**Authors:** Renata Leite Pedreira, Juliana Martins Leal, Keline Jácome Silvestre, Alice Paixão Lisboa, Alexandre Carlos Gripp

**Affiliations:** 1 Universidade do Estado do Rio de Janeiro (UERJ) – Rio de Janeiro (RJ), Brazil

**Keywords:** Acrodermatite, Adolescente, Exantema

## Abstract

Gianotti-Crosti syndrome is a rare disease characterized by acral papular eruption with symmetrical distribution. It is a benign and self-limited disease; the symptoms disappear after two to eight weeks, without recurrences or scars. Skin lesions are usually asymptomatic. Prodrome might occur, suggesting upper respiratory infection, or constitutional symptoms. Diagnosis is eminently clinical, and this disease is associated with viral infections. Due to its rarity and low occurrence in adolescents and adults, we report a case of Gianotti-Crosti syndrome of a teenager.

## INTRODUCTION

Gianotti-Crosti Syndrome (GCS), or papular acrodermatitis of childhood, is a rare and self-limited dermatosis. Its peak incidence occurs in infants between one and six years of age. Clinically, it is characterized by symmetrical papular eruption with acral distribution. The torso generally remains intact, and lesions may be asymptomatic or pruritic. The rash develops abruptly; it may cause prodrome with pharyngitis, infections in the upper airways, and diarrhea.^1-4^ Cutaneous signs and symptoms seem to depend more on the patient’s individual characteristics, rather than on the causative agent.^1,2,3^ Classic findings are multiple monomorphic, and erythematous or normochromic papules, which might be slightly pruritic and confluent. They have acral, symmetrical distribution on the face, on the extensor surfaces of the extremities, and on the gluteal region. The torso, palms and soles are usually spared, but if affected, diagnosis should not be ruled out. Systemic manifestations are unusual and include low-grade fever, generalized lymphadenopathy, hepatomegaly, and splenomegaly. Mucosa and nails are not affected.^1,3,4,5,6^

This disease might be associated with viral infections such as Epstein-Barr (EBV) and herpesvirus type B. GCS prevalence and incidence are unknown. As the lesions may be mistakenly diagnosed as a viral rash, this syndrome is underdiagnosed. In infants GCS has a characteristic manifestation and may be easily diagnosed.

Due to its rare ocurrence in adults, we report a case of GCS in an adolescent patient.

## CASE REPORT

**Figure 1:**
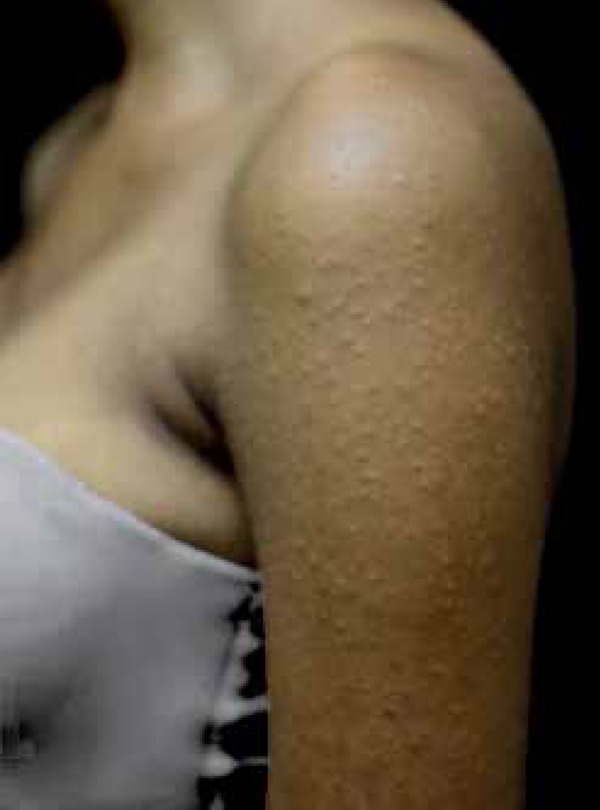
Multiple mon omorphic papules on the extensor surface of the upper limb

**Figure 2:**
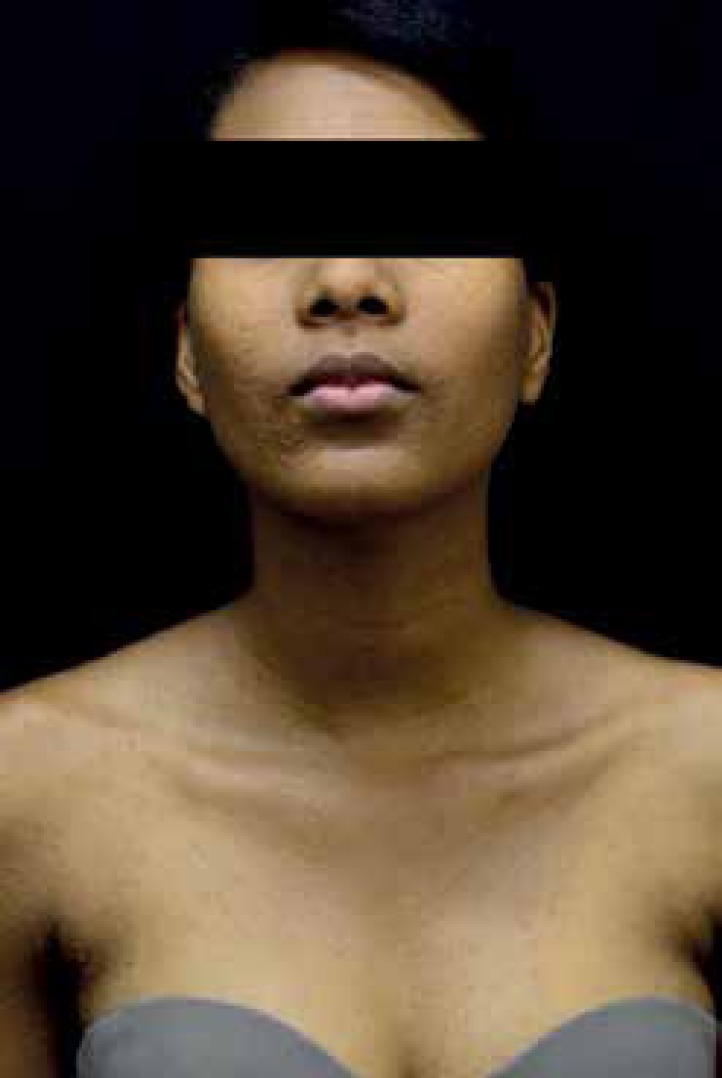
Papular lesions affecting the face and upper torso

**Figure 3:**
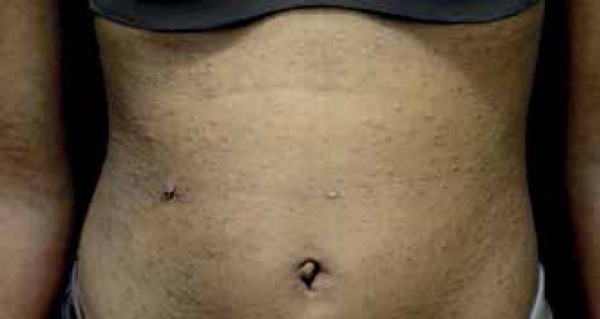
Papular lesions, some of which crusted, in the abdomen and upper limbs

**Figure 4:**
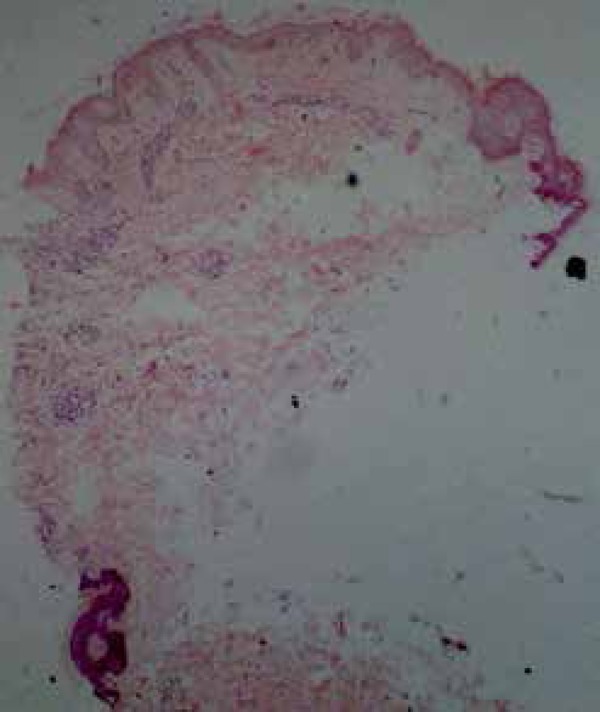
Focal parakeratosis and epidermal spongiosis. Papillary dermal edema and perivascular lymphocytic inflammatory infiltrate in the superficial dermis (40x magnification

An 18-year-old brown-skinned female patient showed acute skin lesions on her upper limbs, which then spread to her face, neck, torso, abdomen, and lower limbs (Figures 1 to 3). She reported mild itching and burning lesions. Dermatological examination showed multiple normochromic and monomorphic papules, some of which crusted, symmetrically distributed. The patient had no history of illness or use of medications. Histopathological findings, although non-specific, were consistent with those described in the syndrome (Figure 4). Laboratory and serology exams were negative. Symptomatic treatment with antihistamines and antipyretics was prescribed. Spontaneous regression occurred after five weeks of evolution, leaving no scars.

## DISCUSSION

GCS was first described in 1955 by Gianotti as a papular, monomorphic, self-limited, symmetrically distributed eruption on the face, buttocks, and extremities. It affects children from two to six years of age.^1,2^

It is described as an infant dermatosis, but rare cases have been reported in adults, mostly females.^7^ This syndrome can be found worldwide, but its impact is unknown, due to its underdiagnosis.^1,3^ GCS affects children of all genders and races, and can be mistakenly diagnosed as a viral rash.^3^

In 1970, it was associated with hepatitis B virus infection.^2^ However, GCS is currently considered a standard-reaction dermatosis associated with viral and bacterial infecions, and immunization.^1,2,5^ Its pathogenesis, including the acral distribution of lesions, is still undefined. It is proposed that viruses or circulating immune complexes were the cause of the cutaneous findings, resulting from delayed hypersensitivity reaction of the cells.^1,5,6^ It was associated with atopic dermatitis and a family history of atopy, but the predisposing mechanism of these patients is unclear.^1,3,4^

Diagnosis is clinical.^1,2,4,7^ It may be atypical in adults; due to its rare occurrence, it should be included in the differential diagnosis for molluscum contagiosum, papular urticaria, drug eruption, and erythema multiforme.^1,3,5^ Changes in liver profile might be caused by hepatitis or EBV.^1,5,6^ Histopathologic findings are non-specific, and include: acanthosis, hyperkeratosis, focal parakeratosis, spongiosis, edema of the papillary dermis with extravasation of erythrocytes, superficial perivascular lymphohistiocytic inflammatory infiltrate, and dilated dermal capillaries.^1,3,7^

The course of disease is benign and self-limited, and its resolution takes up to eight weeks, leaving no scars. Recurrences have been reported, but they are uncommon. Some cases may result in hypochromia or post-inflammatory hyperchromia.^1,2,5,7^

Most cases require no treatment. Lesions seem to be resolved more quickly with the use of medium-potency topical corticoids. Antihistamines may be prescribed to control itching, and systemic corticosteroids are recommended in acute cases.^1,3,6,7^

The publication of new cases and their potential triggering factors shall help elucidate and better understand the pathogenesis of this syndrome. Dermatologists should always be careful and include GCS in the differential diagnosis for rash and papules.
